# Specialized Positioning of Myonuclei Near Cell-Cell Junctions

**DOI:** 10.3389/fphys.2018.01531

**Published:** 2018-11-01

**Authors:** Margherita Perillo, Eric S. Folker

**Affiliations:** Department of Biology, Boston College, Chestnut Hill, MA, United States

**Keywords:** myonuclei, nuclear position, nesprin, KASH, skeletal muscle

## Abstract

Skeletal muscles are large cells with multiple nuclei that are precisely positioned. The importance of the correct nuclear position is highlighted by the correlation between mispositioned nuclei and muscle disease (Spiro et al., 1966; Gueneau et al., 2009). Myonuclei are generally considered to be equivalent and therefore how far nuclei are from their nearest neighbor is the primary measurement of nuclear positioning. However, skeletal muscles have two specialized cell-cell contacts, the neuromuscular (NMJ) and the myotendinous junction (MTJ). Using these cell-cell contacts as reference points, we have determined that there are at least two distinct populations of myonuclei whose position is uniquely regulated. The post-synaptic myonuclei (PSMs) near the NMJ, and the myonuclei near the myotendinous junction myonuclei (MJMs) have different spacing requirements compared to other myonuclei. The correct positioning of pairs of PSMs depends on the specific action of dynein and kinesin. Positions of the PSMs and MJMs relative to the junctions that define them depend on the KASH-domain protein, Klar. We also found that MJMs are positioned close to the MTJ as a consequence of muscle stretching. Our study defines for the first time that nuclei in skeletal muscles are not all equally positioned, and that subsets of distinct myonuclei have specialized rules that dictate their spacing.

## Introduction

Syncytia are multinucleated cells that typically exist as embryonic developmental transitions, such as the fly syncytial blastoderm, or as a terminal differentiated specialized cell, including skeletal muscles, osteoclasts, or placenta. Although much work has determined how these cells become syncytial (Mazumdar and Mazumdar, 2002; Gerbaud and Pidoux, 2015; Kim et al., 2015; Deng et al., 2017), how many nuclei share a common cytoplasm is less well-understood. One fundamental question is whether all of the nuclei in a multinucleated cell are functionally equivalent. If nuclei do indeed have independent functions, each function is likely regulated by the position of the nucleus relative to other nuclei and cellular hallmarks.

In skeletal muscle cells, individual nuclei undergo several nuclear movements that result in peripherally positioned nuclei evenly spread along the surface of the myofiber. The regular positioning of nuclei, and the movements that generate this pattern, are conserved from *Drosophila* to mammals (Folker and Baylies, 2013; Roman and Gomes, 2017). The evolutionary conservation suggests that myonuclear movements are critical to muscle development and function. Furthermore, mispositioned nuclei are abundant in several muscle disorders, including Centronuclear myopaties (CNM), Duchenne muscular dystrophy (DMD), Emery-Dreifuss muscular dystrophy (EDMD), and Fascioscapulohumural muscular dystrophy. Finally, genes that are mutated in patients with EDMD, DMD, CNM, and FSHD all directly impact myonuclear movement (Spiro et al., 1966; Puckelwartz et al., 2009; Zhang et al., 2009; D'Alessandro et al., 2015; Iyer et al., 2016; Collins et al., 2017; Vanderplanck et al., 2018). Collectively, these results suggest that the position of each nucleus is critical to its function.

Myonuclear position is a microtubule-dependent process that requires the plus-end directed motor Kinesin and the minus-end directed motor Dynein (Cadot et al., 2012; Folker et al., 2012; Metzger et al., 2012; Wilson and Holzbaur, 2012, 2014). Mechanistically, Dynein and Kinesin coordinate nuclear movement by two distinct pathways. The cortical pathway relies on Dynein that is stabilized at the cell cortex by Partner of Inscuteable (Pins/Rapsynoid on Flybase). From the cortex, Dynein pulls microtubule minus-ends, and the attached myonuclei toward the cell cortex (Folker et al., 2012). In the proximal pathway, Kinesin and Dynein exert force directly on the nucleus and transport the nucleus as a large vesicle (Wilson and Holzbaur, 2012, 2014; Folker et al., 2014).

Both mechanisms of nuclear movement necessitate interactions between the nucleus and the cytoskeleton. KASH-domain proteins span the outer nuclear membrane and provide the connection between the nucleus and the cytoskeleton (Starr and Han, 2002; Crisp, 2006; Luxton and Starr, 2014). KASH-domain proteins are critical for nuclear movement and position in several cell types including skeletal muscle (Fridolfsson et al., 2010; Elhanany-Tamir et al., 2012; Wilson and Holzbaur, 2014; Collins et al., 2017). Although the KASH-domain proteins, Dynein, and Kinesin regulate myonuclear movements in mammalian cultures and in *Drosophila*, it has not been investigated whether all nuclei are equally sensitive to the activities of these proteins.

Muscles have two distinct cell-cell contacts that may necessitate specialized nuclear positioning, the neuromuscular junction (NMJ) and the myotendinous junction (MTJ). In several vertebrate organisms, myonuclei cluster underneath the NMJ (Englander and Rubin, 1987; Sanes et al., 1991; Grady et al., 2005). However, it is not known whether clustering of postsynaptic myonuclei (PSMs) is conserved in *Drosophila*. The MTJ is the contact between a muscle cell and tendon cell through which force is transmitted during muscle stretching and contraction (Tidball, 1991; Weitkunat et al., 2014; Valdivia et al., 2017). No unique positioning of nuclei at the MTJ has been described (Bruusgaard et al., 2004), however the possibility has not been rigorously tested.

Here we demonstrate that nuclei near the NMJ have unique spatial requirements compared to most myonuclei. Furthermore, certain populations of myonuclei are uniquely sensitive to the disruption of genes that regulate nuclear position. Specifically, Dynein and Kinesin act to regulate the distance between myonuclei near the NMJ, whereas Klar regulates the distance between myonuclei and the NMJ. Finally, Klar also regulates the position of nuclei relative to the MTJ, and the positional response of those nuclei to mechanical stimulus. Overall, these data define for the first time that nuclei in skeletal muscles are not all equally positioned, and that different populations respond differently to the depletion of proteins that move nuclei.

## Results

### Identification of subsets of nuclei

**Figure 1:**
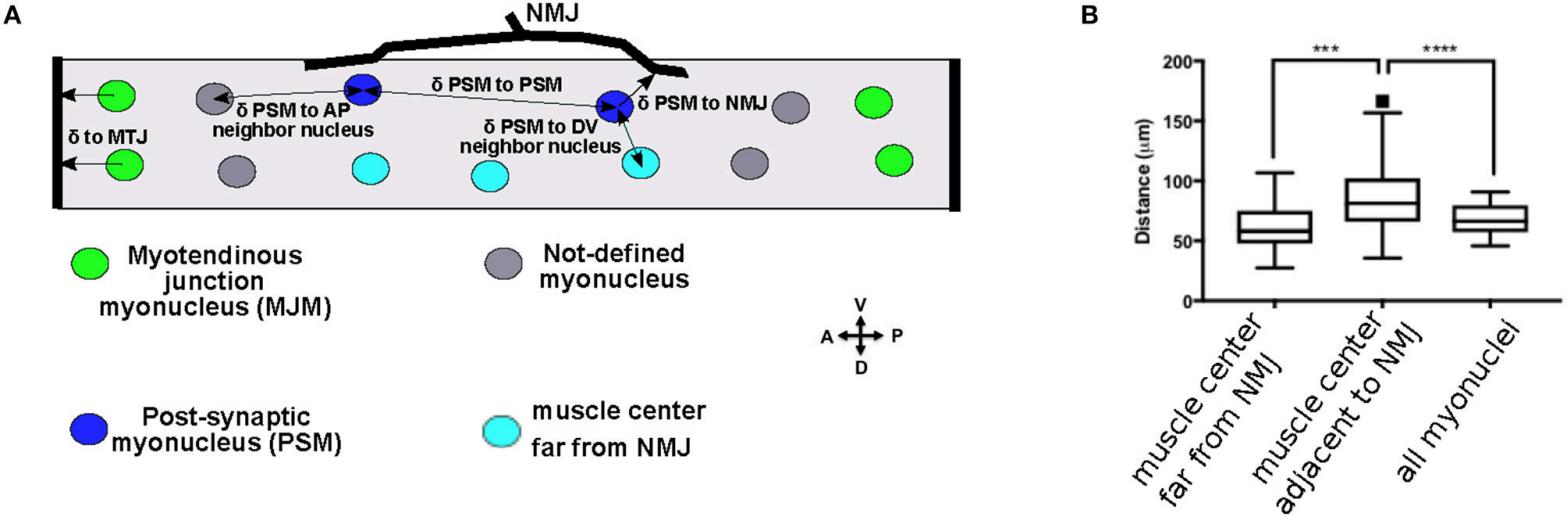
Subsets of myonuclei are defined by their proximity to cell-cell contacts. **(A)** Cartoon of a 3rd instar larval muscle 6. Post-synaptic myonuclei (PSM) are shown in blue, myotendinous junction myonuclei (MJM) are shown in green, and the other undefined myonuclei are shown in gray. Black arrows indicate the measurements described in the text. **(B)** Tukey boxplots indicating the distance to the nearest nucleus for nuclei positioned in the muscle center, but on the opposite side from the NMJ; in the muscle center adjacent to the NMJ, or for all myonuclei. ^***^p < 0.0005, ^****^p < 0.0001.

To determine whether all nuclei in a myofiber are positioned by the same mechanisms, it was first necessary to determine whether distinct populations of nuclei exist within the syncytial myofiber. We dissected 3rd instar *Drosophila* larvae and measured the positions of the nuclei in abdominal muscle 6 because the entire muscle is easily visible after dissection. In controls, nuclei were positioned in two parallel rows along the anterior-posterior (A-P) axis of the muscle fiber (Figure 1). In previous studies, all nuclei were treated as equal, and a single value of average internuclear distance was reported for each muscle (Elhanany-Tamir et al., 2012; Folker et al., 2012; Metzger et al., 2012; Schulman et al., 2014; Collins et al., 2017). Here, we specifically measured the position of nuclei relative to two specialized cell-cell contacts, the NMJ and the MTJ.

**Figure 2:**
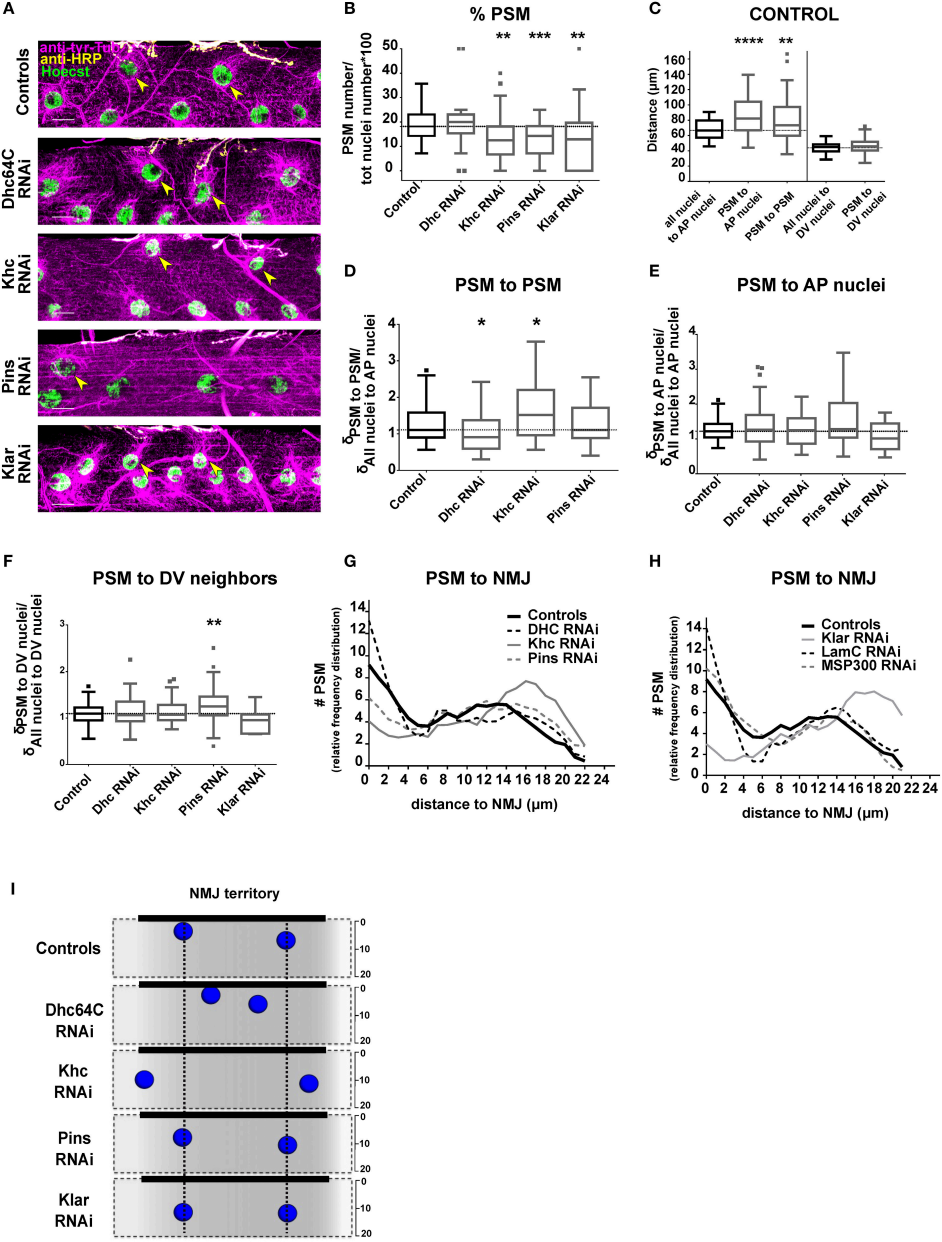
Positioning of PSMs in the NMJ territory. **(A)** Max intensity projections of confocal images of the central section from a larval muscle near the NMJ in the indicated genotypes. Microtubules, magenta; Nuclei, green; NMJ, yellow. Yellow arrowheads mark the PSMs. Scale bar = 20 μm. **(B)** Tukey boxplots indicating the percentage of total nuclei that are PSMs in indicated genotypes. **(C)** Tukey boxplots showing the distance between specific sets of myonuclei in controls. On the A-P axis, postsynaptic myonuclei (PSMs) are further from their neighbors when compared to the other nuclei. **(D)** Tukey boxplots indicating the distance between neighboring PSMs (when more than 1 PSM per muscle can be counted). The distance is normalized to the average distance between all myonuclei and their nearest neighbor on the AP axis for the entire muscle. **(E,F)** Tukey boxplots showing the distance between PSMs and their nearest neighbors on the A-P axis **(E)** or the distance between a PSM and the nearest neighbor on the D-V axis **(F)** for the indicated genotypes. Distances were normalized to the average inter-nuclear distance for all nuclei on the same axis within the same muscle. **(G,H)** Graphs indicating the distribution of distances between PSMs and the NMJ. Student's *t*-test was used for comparison to controls (indicated as a black dashed line). In all boxplot graphs, controls are in black and the other genotypes are in gray. **(I)** Summary of the data shown in **(A–H)**. Nuclei represent the average nuclear positions. Vertical black dotted lines indicate PSM position in controls. Student's *t*-test was used for comparison to controls; ^*^p < 0.05, ^**^p < 0.005, ^***^p < 0.0005, ^****^p < 0.0001.

In vertebrates, several nuclei, called post-synaptic myonuclei (PSMs), cluster near the NMJ (Grady et al., 2005). However, there is no obvious clustering of nuclei near the NMJ in *Drosophila* larval muscles. To determine whether the nuclei nearest the NMJ had a distinct spacing compared to other nuclei in the muscle fiber, we measured the distances between each nucleus and its nearest neighbor on the A-P axis because nuclei were positioned in two parallel rows on the A-P axis. Compared to all other nuclei, the nuclei adjacent to the NMJ were further from their nearest neighbor (Figure 1B). Because the NMJ is near the center of the muscle, this difference could be a consequence of their central position rather than their being adjacent to the NMJ. Therefore, we measured the internuclear distance for the nuclei near the center of the muscle, but on the side opposite from the NMJ. These nuclei were closer to their nearest neighbor than were the nuclei adjacent to the NMJ. Thus, the nuclei that are nearest to the NMJ have distinct spacing requirements compared to other myonuclei in *Drosophila*. We therefore defined PSMs as myonuclei that were positioned between 0 and 20 μm from the NMJ (the diameter of one myonucleus) (Figure 1, blue nuclei and Figure 2A).

### Post-synaptic myonuclei (PSMs) are spatially different from the other myonuclei

We counted the number of PSMs in control 3rd instar larvae, and found that muscles had between 1 and 4 PSMs (Supplementary Figure 1), accounting for between 7 and 35% of total myonuclei in the myofiber (Figure 2B). Closer inspection revealed that there were two subpopulations of PSMs (Figures 2G,H). One population of PSMs was between 0 and 5 μm from the nearest bouton and accounted for 36.52% of total PSMs. The second population of PSMs was between 5 and 20 μm away from the nearest bouton, and accounted for 63.47% of the total PSMs.

We then measured the distance between PSMs and specific sets of neighbor nuclei, including their nearest neighbors on either the A-P axis (on which PSMs have both non-PSM and PSM neighbors) or the D-V axis (on which PSMs only have non-PSM neighbors, positioned dorsally). In controls, the distance between PSMs and their nearest non-PSM neighbor on the A-P axis was greater than the distance between two non-PSMs (Figure 2C). Additionally, the distance between two PSMs was greater than average internuclear distance for all nuclei (Figure 2C). However, the distance between PSMs and their nearest neighbor on the D-V axis was similar to the D-V spacing of all other nuclei (Figure 2C). These data further suggest that the PSMs have unique spacing requirements on the A-P axis of the muscle compared to the majority of myonuclei.

### Dynein and kinesin regulate nuclear position near the NMJ

To determine the factors necessary for the unique positioning of PSMs, we depleted genes that regulate general nuclear position in the developing *Drosophila* muscle (Elhanany-Tamir et al., 2012; Folker et al., 2012; Metzger et al., 2012). To exclude the potential influence of the motor neuron on the position of myonuclei, we expressed RNAi specifically in muscle using the *Dmef2-GAL4* to drive the expression of UAS-RNAi (Brand and Perrimon, 1993).

We first depleted the microtubule motors, Dynein and Kinesin, which provide the force to move myonuclei. Because depletion of either Dynein or Kinesin disrupted general myonuclear position (Folker et al., 2012; Metzger et al., 2012; Wilson and Holzbaur, 2012), we measured PSM position as a function of general nuclear spacing throughout the myofiber (see Materials and Methods section). Kinesin depletion and Dynein depletion had opposite effects on the distance between PSMs. In Dynein-depleted muscles, PSMs were closer together when compared to controls (Figures 2A,D,I). In Kinesin-depleted muscles, PSMs were further apart when compared to controls (Figures 2A,D,I). Although the distance between two PSMs was sensitive to the depletion of either Dynein or Kinesin, the distance between a PSM and a non-PSM was not (Figures 2E,F). These data suggest that the position of PSMs, relative to other PSMs, is coordinated by a balance of Dynein and Kinesin activity.

We next tested whether Pins (partner of inscuteable, Rapsynoid on Flybase), which recruits and stabilizes Dynein at the cortex, and is important for general positioning of myonuclei (Folker et al., 2012), has any specific effects on PSM position. Pins depletion did not affect the distance between pairs of PSMs or the distance between PSMs and their non-PSM neighbor on the A-P axis (Figures 2A,D,E,I). However, PSMs were further from their nearest neighbor on the D-V axis compared to controls (Figure 2F). These data suggest that the D-V spacing of PSMs is particularly sensitive to Pins expression. All together, these data demonstrate that Dynein, Kinesin and Pins have unique impacts on the positioning of the PSMs compared to other myonuclei.

### Klarsicht regulates the distance between PSMs and the NMJ

Having found that the distance between PSMs is regulated by Dynein and Kinesin, we tested whether the distance between PSMs and the NMJ is also regulated by these two microtubule motors. In Dynein-depleted muscles the number of PSMs (Figure 2B), and the distances between PSMs and the NMJ were similar to controls (Figures 2A,G,I). In Kinesin-depleted muscles the total number of PSMs was reduced (Figure 2B). Furthermore, the number of PSMs within 5 μm of the NMJ (close PSMs) was decreased, and the number of PSMs between 5 and 20 μm from the NMJ (far PSMs) was increased (Figures 2A,G,I). Pins depletion reduced the percentage of myonuclei that were PSMs (Figure 2B), and increased the percentage of PSMs that were far from the NMJ (Figures 2A,H). These data suggest that Kinesin and Pins are essential for positioning PSMs close to the NMJ (within 5 μm).

To position nuclei, the cytoskeletal factors that generate force must interact with the nucleus. KASH-domain proteins span the outer nuclear envelope and facilitate the direct interaction between the nucleus and the cytoskeleton, and are necessary to transmit force from the cytoskeleton to the nucleoskeleton (Starr and Han, 2002; Luxton et al., 2010; Guilluy et al., 2014) that is composed of nuclear lamins. Both KASH-domain proteins and the nuclear lamin, LamC (Lamin A/C in mammals), are necessary for proper nuclear positioning in muscle (Dialynas et al., 2010; Folker et al., 2011; Elhanany-Tamir et al., 2012; Zwerger et al., 2013). Therefore, we tested whether positioning of PSMs was dependent on the *Drosophila* KASH proteins (Klar and Msp300) and LamC. In muscles depleted of Klar, the total number of PSMs (Figure 2B) and the percentage of PSMs that were within 5 μm of the NMJ were reduced (Figures 2A,H,I). There was no change in the number or position of PSMs in Msp300-depleted or LamC-depleted animals (Figure 2H). Thus, the KASH-domain protein Klar is specifically required to position nuclei within 5 μm of the NMJ.

### Klar and pins position myonuclei near the MTJ

**Figure 3:**
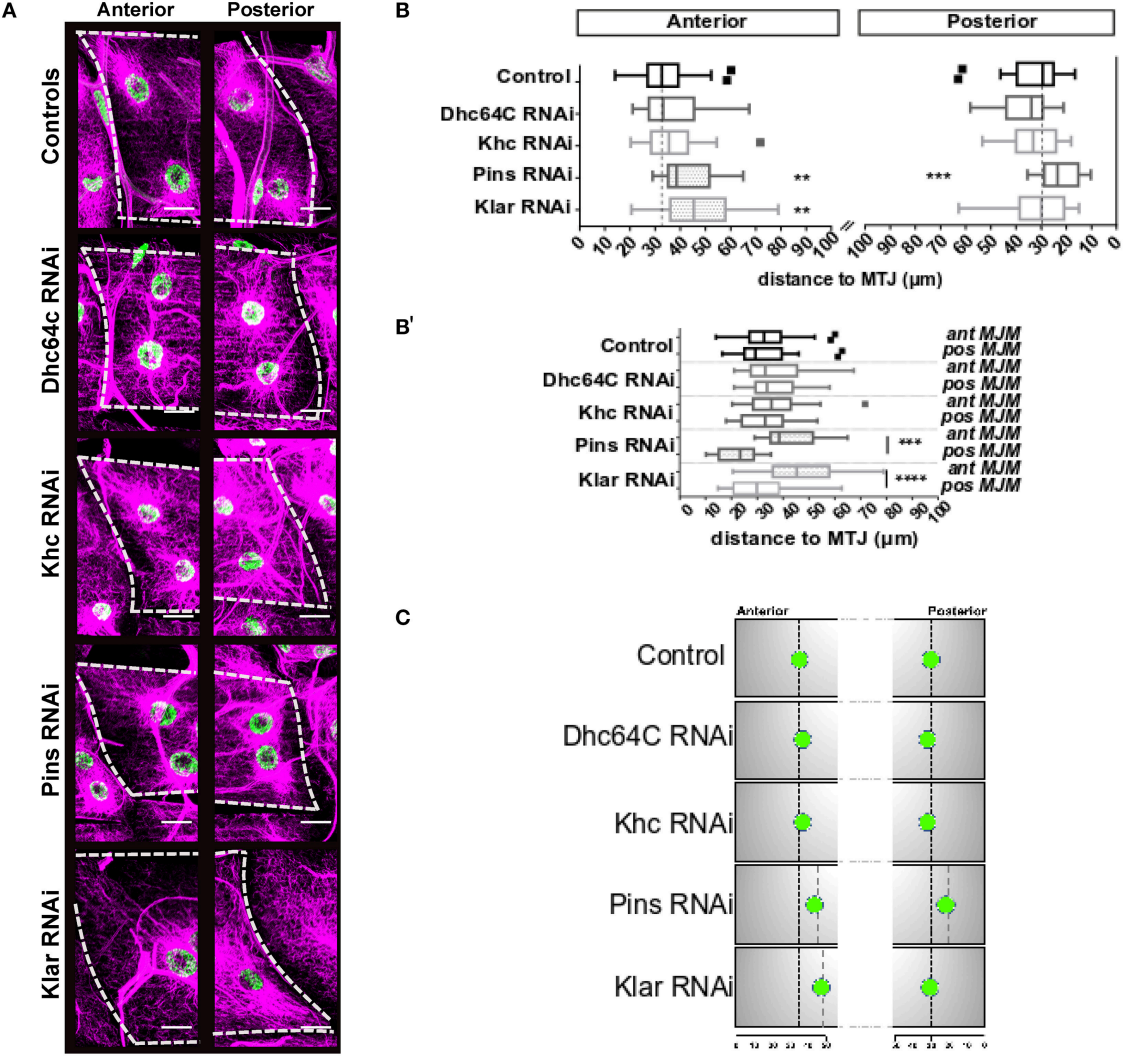
Distance between MJMs and the MTJ. **(A)** Max intensity projections of confocal images of myotendinous regions of the indicated genotypes. Microtubules, magenta; nuclei green. Scale bar = 20 μm **(B)** Tukey boxplots showing the distance between MJMs and the MTJ for the indicated genotype in segments A3 (top) and A4 (bottom). **(B**′**)** Same data as in **(B)**, but with anterior and posterior MJMs grouped vertically. Anterior MJMs are shown on the left and posterior MJMs are shown on the right. Vertical dashed black lines indicate the median value for the controls. **(C)** Summary of the data shown in **(A,B)**. For sake of simplicity, only one MJM is represented for each MTJ. Nuclei represent the average nuclear positions. Vertical black dotted lines indicate MJM position in controls. Gray dotted lines indicate position of MJMs that are significantly different from controls. Student's *t*-test was used for comparison to controls; ^**^p < 0.005, ^***^p < 0.0005, ^****^p < 0.0001.

The myotendinous junction (MTJ) is another specialized cell-cell contact that may specify a unique subset of myonuclei. To test this, we measured the positions of MTJ myonuclei (MJMs). We defined MJMs as the nuclei in each row that are nearest to either the anterior or posterior MTJ (Figure 1). In controls (Figures 3A,B), the anterior and posterior MJMs were the same distance from the MTJ.

We next depleted genes necessary for general myonuclear positioning and measured the position of MJMs. Neither Kinesin-depletion nor Dynein-depletion had any impact on the position of MJMs relative to the MTJ (Figures 3A,B). Klar-depletion had no effect on the position of posterior MJMs (Figures 3A,B) but caused the anterior MJMs to be further from the MTJ compared to controls (Figures 3A,B). Pins-depletion also caused anterior MJMs to be further form the MTJ. Additionally, in Pins-depleted animals, the posterior MJMs were closer to the MTJ (Figures 3A,B). Together, these data indicate that the anterior MJMs are particularly sensitive to the expression of Klar and Pins (results are summarized in Figure 3C).

### Nuclei move closer to the MTJ in response to mechanical stimuli

**Figure 4:**
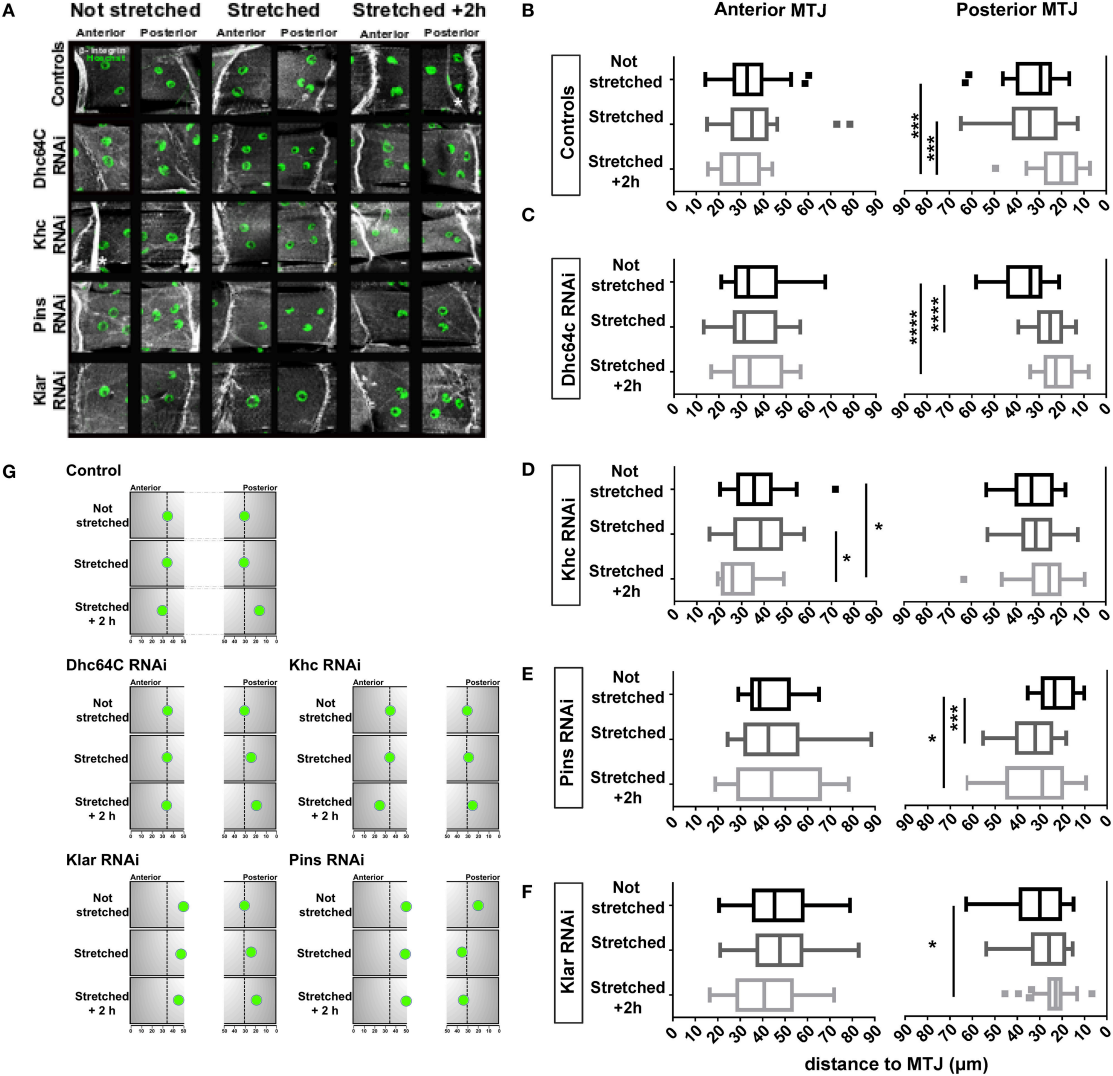
Effects of larval stretching on MJM positioning. **(A)** Max intensity projections of confocal images of anterior and posterior MTJs for the indicated genotypes stained for β-integrin (gray) and nuclei (green). White asterisks mark trachea branches, which are not part of the muscle. Scale bar is 10 μm **(B-F)** Tukey boxplots showing the distance between MJMs and the MTJ in larval that have not been stretched (not stretched), larvae dissected immediately after stretching (stretched), larva that have been stretched and dissected after a 2 h recovery (stretched +2 h). **(G)** Cartoon of MJM position in stretching experiments. Summary of the stretching experiments from **(A-F)**. For sake of simplicity, only one MJM is represented for each MTJ. Black arrows indicate the direction of the change in nuclear positioning. ^*^p < 0.05, ^***^p < 0.0005, ^****^p < 0.0001.

Because the MTJ is susceptible to damage during stretch or eccentric contractions (Tidball, 1991; Frenette and Côté, 2000), nuclear position may change as a consequence of mechanical stress. We measured the position of MJMs before and after muscle stretching to determine whether nuclei moved in response to a mechanical stimulus. Third instar larvae were stretched for 10 min, then dissected either immediately after stretching (time = 5 min) or after a 2-h recovery. We measured the distance between the MTJ and the MJMs at both the anterior and posterior end of the muscle (Figures 4A,B). In control muscles, stretching did not immediately change the position of the MJMs at either the anterior of posterior muscle end (Figures 4A,B and cartoon in Figure 4G; statistics is in Supplementary Table 3). After a 2-h recovery, posterior MJMs were closer to the MTJ (Figures 4A,B, and cartoon in Figure 4G). These data suggest that MJMs are anchored in position, but that stretching triggers a response that moves MJMs closer to the MTJ.

We next tested whether the proteins necessary for positioning nuclei near the MTJ also regulate the movement of MJMs following muscle stretching. Although MJMs in Klar-depleted muscles were positioned further from the MTJ before stretching, their behavior in response to stretching was similar to controls. Anterior MJMs did not change position after stretching whereas posterior MJMs were closer to the MTJ 2 h after stretching (Figures 4A,F). Although nuclei moved in the same direction, the degree of movement was reduced in Klar-depleted muscles compared to controls. In Pins-depleted muscles, posterior MJMs were significantly further from the MTJ immediately after stretching (Figures 4A,E). After a 2-h recovery, posterior MJMs were still further from MTJ (Figures 4A,E). In both Klar- and Pins-depleted animals, only the posterior MJMs changed position after a mechanical stimulus. Because the posterior MJMs were closer to the MTJ before stretching, these data may suggest that nuclear movement as a response to stretching may be dependent on the initial nuclear position.

To determine the impact of the cytoskeleton on stretch-induced nuclear movement, we depleted Dynein and Kinesin and measured nuclear position after stretching. In Dynein-depleted muscles, anterior MJMs did not change position 2 h after stretching, but posterior MJMs were closer to the MTJ immediately after stretching (Figures 4A,C). Being already close to the MTJ, posterior MJMs maintain their position at 2 h after stretching. These data suggests that Dynein functions to maintain MJMs in place during stretching. The nuclei in Kinesin-depleted muscles responded to mechanical stress. However, in these muscles, the anterior nuclei moved closer to the MTJ and the posterior nuclei maintained their position (Figures 4A,D,G).

## Discussion

In vertebrates, myonuclei at the post-synaptic site are clustered near the NMJ (Englander and Rubin, 1987; Grady et al., 2005). However, the mechanism that underlies nuclear spatial commitment, and whether non-uniform nuclear distribution is conserved, is not known. Using *Drosophila* as a model organism, we found that PSMs are further from their nearest neighbor compared to all other myonuclei demonstrating that unique positioning of PSMs is conserved. More broadly, these data suggest that each nucleus may have specialized spatial requirements.

The proteins that position PSMs are the same as those that position all myonuclei. However, disrupting expression of these proteins has distinct effects on PSMs. Depletion of Kinesin and Dynein had opposite effects on the distance between PSMs suggesting that the distance between PSMs is regulated by microtubules and may require a balance of forces similar to those necessary to maintain a mitotic spindle (Gaglio, 1996). The effect of Kinesin-depletion is surprising given that in general, Kinesin-depletion causes nuclei to cluster in muscle. Thus, the requirements and the mechanisms of positioning nuclei near the NMJ are unique compared to those for general nuclear positioning.

Additionally, Kinesin and the KASH-domain protein Klar regulate the distance between the NMJ and the PSMs. That Dynein is not involved suggests that cortical pulling does not regulate the final distance between a nucleus and the NMJ. This suggests a Kinesin- and Klar-dependent mechanism, similar to the mechanisms that drive lipid droplet transport in embryos and nuclear migration in the developing eye (Welte et al., 1998; Mosley-Bishop et al., 1999), moves PSMs to their the final position.

Prior to this work, the position of nuclei relative to the MTJ had not been extensively analyzed. One study showed that in mouse extensor digitorum longus muscles, myonuclei at the MTJ did not show a unique distribution, unless the fiber was undergoing repair or growth which involved the incorporation of additional nuclei (Bruusgaard et al., 2004). However, that study did not provide measurements or genetic regulators. Here we measured for the first time the position of MJMs, and found that these nuclei were 25–30 μm from the MTJ and that the positioning of myonuclei near the MTJ was Klar-dependent and Pins-dependent. Thus, Klar (on the nuclear envelope), and Pins (at the cell cortex) are required for the precise positioning of nuclei near both specialized cell-cell junctions.

Perhaps the most surprising result of this work is that myonuclei were positioned closer to the MTJ after the larva was stretched (results are summarized in Figure 4G). That the movement of nuclei was not immediate, but required a 2-h recovery indicates that the movement is an active response to stretching. Force transmission at the MTJ is supported by a high concentration of cytoskeletal and structural proteins, and regional synthesis of contractile proteins happens at the MTJ when muscles are stretched (Dix, 1990). Thus, MJMs may be moved toward the MTJ to direct local translation of structural proteins for repair.

Because MJMs did not change position immediately after stretching, they are likely anchored in position. Anchoring of myonuclei is dependent on many of the factors that move nuclei during embryonic muscle development. Depletion of Pins caused MJMs to move away from the MTJ immediately after stretching whereas depletion of Dynein caused nuclei to move closer to the MTJ immediately after stretching. Thus, the anchoring of MJMs is Dynein- and Pins-dependent.

Here, we have shown that not all nuclei are positioned at an equal distance from their neighbors. First, PSMs at the NMJ are positioned such that the gap between PSMs and their neighbors is greater than for other myonuclei. The distance between pairs of PSMs is dependent on Dynein and Kinesin, while the distance between a PSM and the NMJ depends on Kinesin and Klar. Second, we found that the distance between MJMs and the MTJ is regulated by Klar and Pins. Last, we showed for the first time that nuclei are positioned closer to the MTJ after muscle stretching. Our identification of unique subsets of myonuclei indicates that simple analysis of the average distance between myonuclei is insufficient to understand how different genes regulate nuclear position. Furthermore, because nuclear position is aberrant in many muscle disorders, either directly or as a consequence of ongoing muscle repair, this work opens new possibilities toward understanding the role that subsets of nuclei have in such disorders.

## Materials and methods

### *Drosophila* genetics

The following stocks were grown under standard conditions at 25°C: UAS-Dhc64C RNAi (36698; Bloomington *Drosophila* Stock Center [BDSC], Bloomington, IN), UAS-Khc RNAi (35770; BDSC), UAS-Pins RNAi (53968; BDSC), UAS-Klar RNAi (36721; BDSC), UAS-MSP300 RNAi (32377; BDSC), UAS-LamC RNAi (31621; BDSC), UAS-mCherry RNAi (35785; BDSC). UAS-RNAi constructs were driven specifically in the muscle using *DMef2- GAL4*. In all experiments, the mother carried the Gal4 and the father carried the UAS-RNAi.

### Immunohistochemistry

Larvae were dissected as previously described (Collins et al., 2017; Camuglia et al., 2018). Briefly, larvae were dissected in ice-cold 1,4-piperazinediethanesulfonic acid (PIPES) dissection buffer containing 100 mM PIPES (P6757; Sigma- Aldrich), 115 mM d-sucrose (BP220-1; Fisher Scientific), 5 mM trehalose (182550250; Acros Organics), 10 mM sodium bicarbonate (BP328-500; Fisher Scientific), 75 mM potassium chloride (P333- 500; Fisher Scientific), 4 mM magnesium chloride (M1028; Sigma- Aldrich), and 1 mM ethylene glycol tetraacetic acid (28-071-G; Fisher Scientific) and then fixed with 4% Formaldehyde in PIPES buffer (BP531-500; Fisher Scientific).

Antibodies for larva filet staining were used at the following final dilutions: rat anti- tyrosinated tubulin, 1:400 (MAB1864; Millipore), mouse anti-myospheroid (β-integrin), 1:100 (CF.6G11, Developmental Studies Hybridoma Bank). Larval NMJs were labeled with Alexa-Fluor 647-conjugated goat anti-HRP, 1:500 (123-605-021,Jackson ImmunoResearch Laboratories). We used Alexa Fluor 488- and Alexa Fluor 555- conjugated fluorescent secondary antibodies (1:400; Life Technologies), and Hoechst 33342 (1 μg/ml, ThermoFisher). Larvae were mounted in ProLong Gold (P36930; Life Technologies).

All images were acquired on a Zeiss 700 LSM using an Apochromat 40×/1.4 numerical aperture (NA) objective.

### Stretching experiments

Intact third instar larva 4–4.5 mm long were put on a dissection plate and held from both the head and the tail using a vacuum system. Larvae were then stretched along their A-P axis for 10 min, dissected immediately and fixed for immunohistochemistry or put on agar plates for 2 h before dissection. Larval length increased 2 mm during stretching. Stretching was visually monitored through a stereomicroscope. Control larvae were larvae form the same lay pot and of the same size that had not been stretched.

### Analysis of nuclear position

We focused our analysis on muscle 6 (ventral longitudinal muscle 3) because this muscle is at the surface in the dissected larvae and the nuclei are stereotypically arranged in two parallel rows along the length of the muscle. We defined PSMs as myonuclei that are positioned between 0 and 20 μm from the NMJ (the diameter of one myonucleus), measuring the distance between the center of a nucleus and the nearest NMJ branch. The distance between PSMs and their neighbor nuclei was compared to the distance between all myonuclei and their nearest neighbor: *All nuclei to AP neighbor* = distance (μm) of all not-defined myonuclei to their closest nucleus on the AP axis; *PSM to AP neighbor* = distance (μm) of PSM to their closest neighbor nucleus on the AP axis; *PSM to PSM neighbors* = distance of PSM to its closest PSM neighbor, that is always on the AP axis; *All nuclei to DV neighbor* = distance (μm) of all not-defined myonuclei to their closest nucleus on the DV axis; *PSM to DV neighbor* = distance (μm) of PSM to their closest neighbor nucleus on the DV axis. The internuclear distance was measured by using the segmented line tool on ImageJ software. We measured the distance from the center of each nucleus to the center of its nearest nuclear neighbor. The distance of MJM to the MTJ was measured from the center of each MJM to the closest MTJ edge.

### Statistics

All statistics were performed using Prism 4.0 (Graphpad). Graphs (Figures 2, 4, all graphs) are depicted as Tukey boxplots (box extend from 25th to 75th percentiles) and include values of single nuclear distance measurements. Horizontal dotted gray line in the box indicates the median for the control. Bars indicate the max and min value. Values greater than the 75th percentile plus 1.5 times the interquartile distance are represented as single points. For normal distributions, student's *t*-test was used for comparison to controls; ^*^p < 0.05, ^**^p < 0.005, ^****^p < 0.0001. We used the Mann–Whitney test when the data did not follow a Gaussian distribution. Details of statistical analysis are included in Supplementary Tables 1–3.

## Author contributions

MP designed, performed, and analyzed the experiments. EF conceived the project and designed experiments. MP and EF wrote the manuscript.

### Conflict of interest statement

The authors declare that the research was conducted in the absence of any commercial or financial relationships that could be construed as a potential conflict of interest.

## Supplementary material

Supplementary Figure 1 PSM number. Tukey plot that show the number of PSMs as row values (not normalized for total myonuclei/muscle as in Figure 2A). Control is in black. Student's *t*-test was used for comparison to controls; ^**^*p* < 0.005, ^****^*p* < 0.0001.

Supplementary Table 1 Statistical analysis for PSM positioning. Note that *n* numbers represent number of muscles. Since in Klar RNAi only a few muscles had PSMs, it was not possible to analyze PSM to PSM distance. Student's *t*-test was used for comparison to unstretched muscles for each genotype.

Supplementary Table 2 Statistical analysis for MJM positioning. Student's *t*-test was used for comparison to unstretched muscles for each genotype. NP indicates that the non-parametric Mann–Whitney test was used.

Supplementary Table 3 Statistical analysis for nuclear positioning in stretching experiments for segments A3 and A4. Student's *t*-test was used for comparison to unstretched muscles for each genotype. NP indicates that the non-parametric Mann–Whitney test. ^*^*p* < 0.05, ^**^*p* < 0.005, ^***^*p* < 0.0005, ^****^*p* < 0.0001.
