# Supplementary figures and images for: Specialized Positioning of Myonuclei Near Cell-Cell Junctions
Source: Front Physiol. 2018 Nov 1;9:1531. doi: 10.3389/fphys.2018.01531 (PMC6221937; doi:10.3389/fphys.2018.01531)

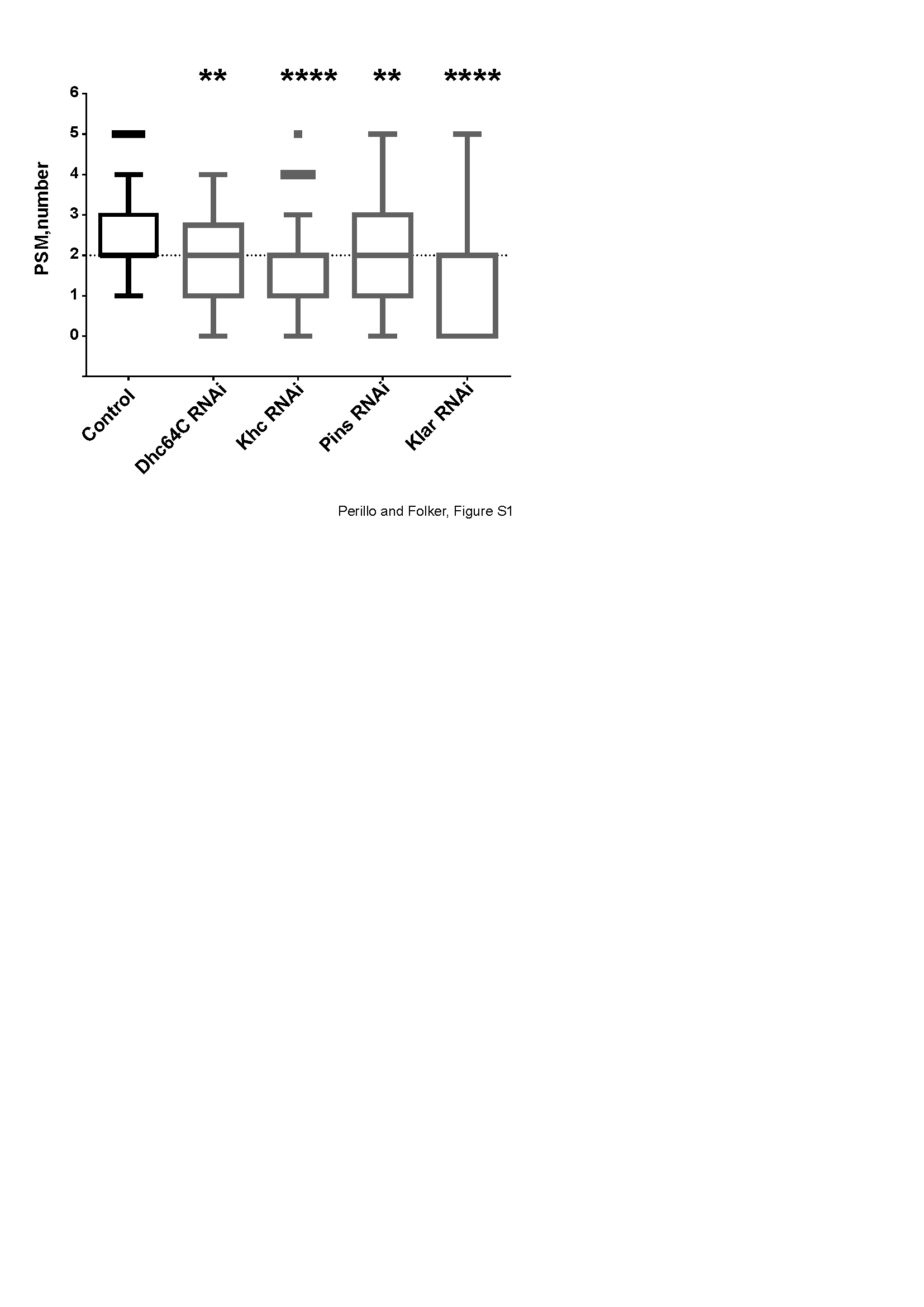

Supplement: Supplementary Figure 1 — PSM number. Tukey plot that show the number of PSMs as row values (not normalized for total myonuclei/muscle as in Figure 2A). Control is in black. Student's t-test was used for comparison to controls; **p < 0.005, ****p < 0.0001. [file Image_1.TIFF]

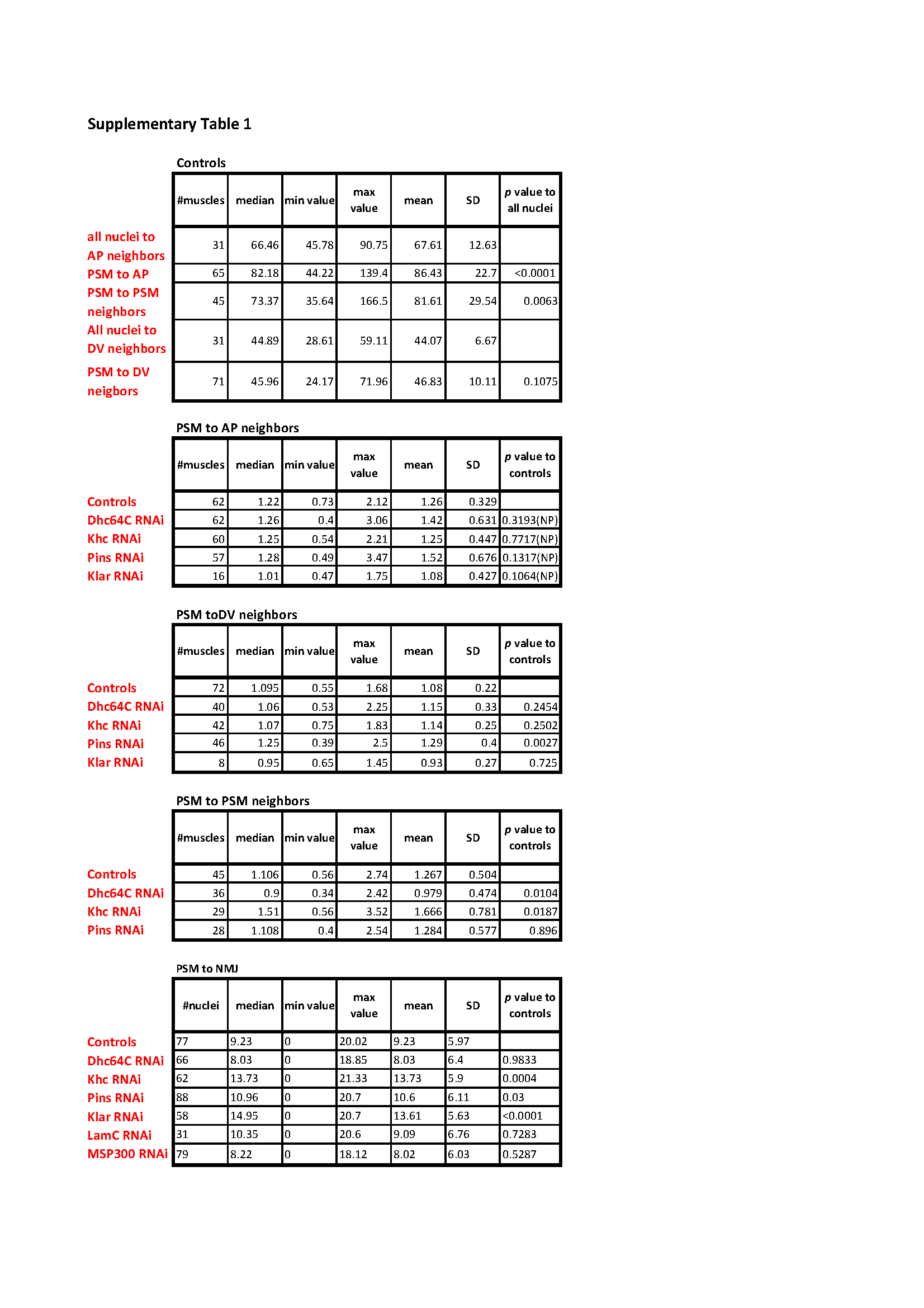

Supplement: Supplementary Table 1 — Statistical analysis for PSM positioning. Note that n numbers represent number of muscles. Since in Klar RNAi only a few muscles had PSMs, it was not possible to analyze PSM to PSM distance. Student's t-test was used for comparison to unstretched muscles for each genotype. [file Image_2.TIFF]

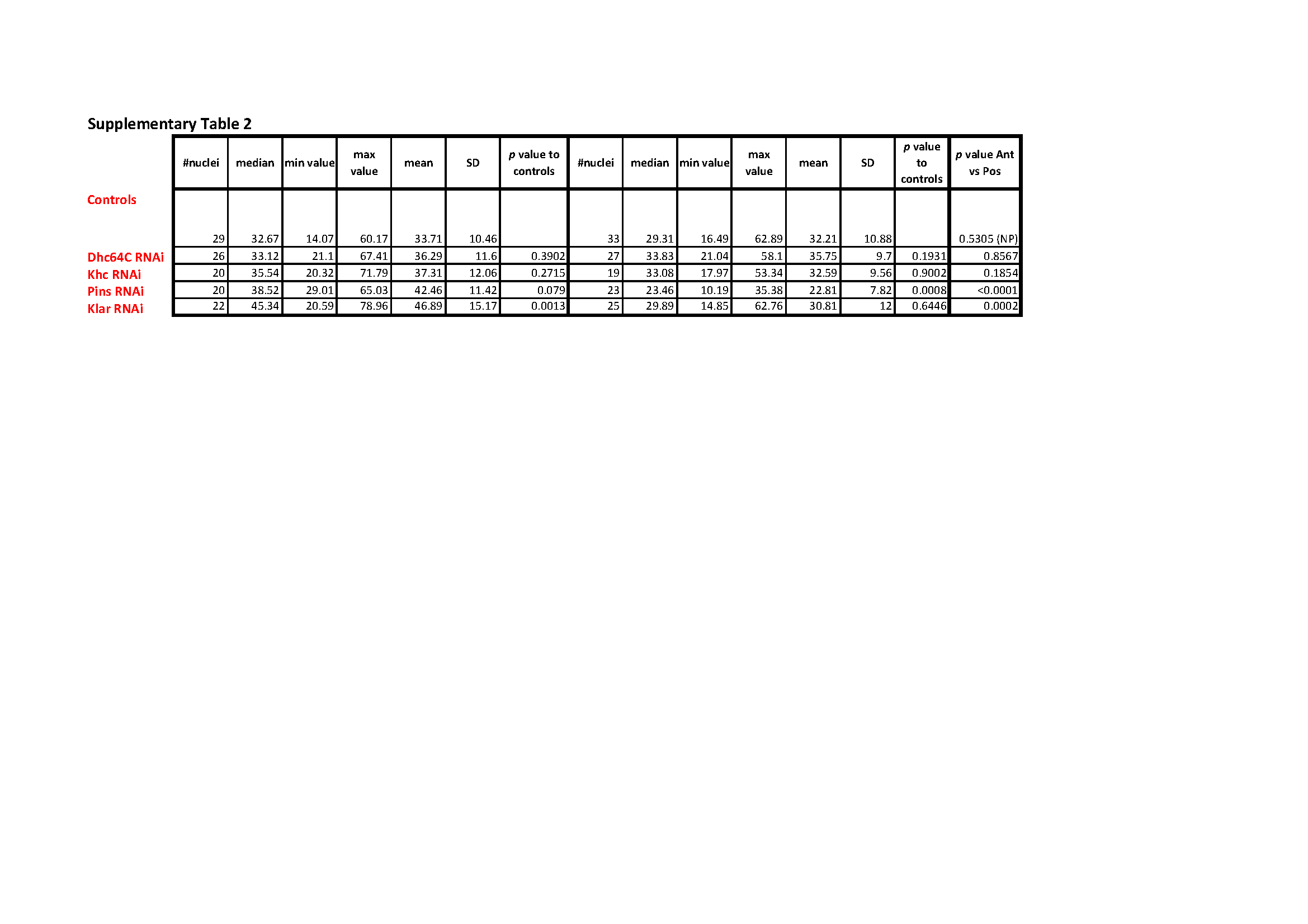

Supplement: Supplementary Table 2 — Statistical analysis for MJM positioning. Student's t-test was used for comparison to unstretched muscles for each genotype. NP indicates that the non-parametric Mann–Whitney test was used. [file Image_3.TIFF]

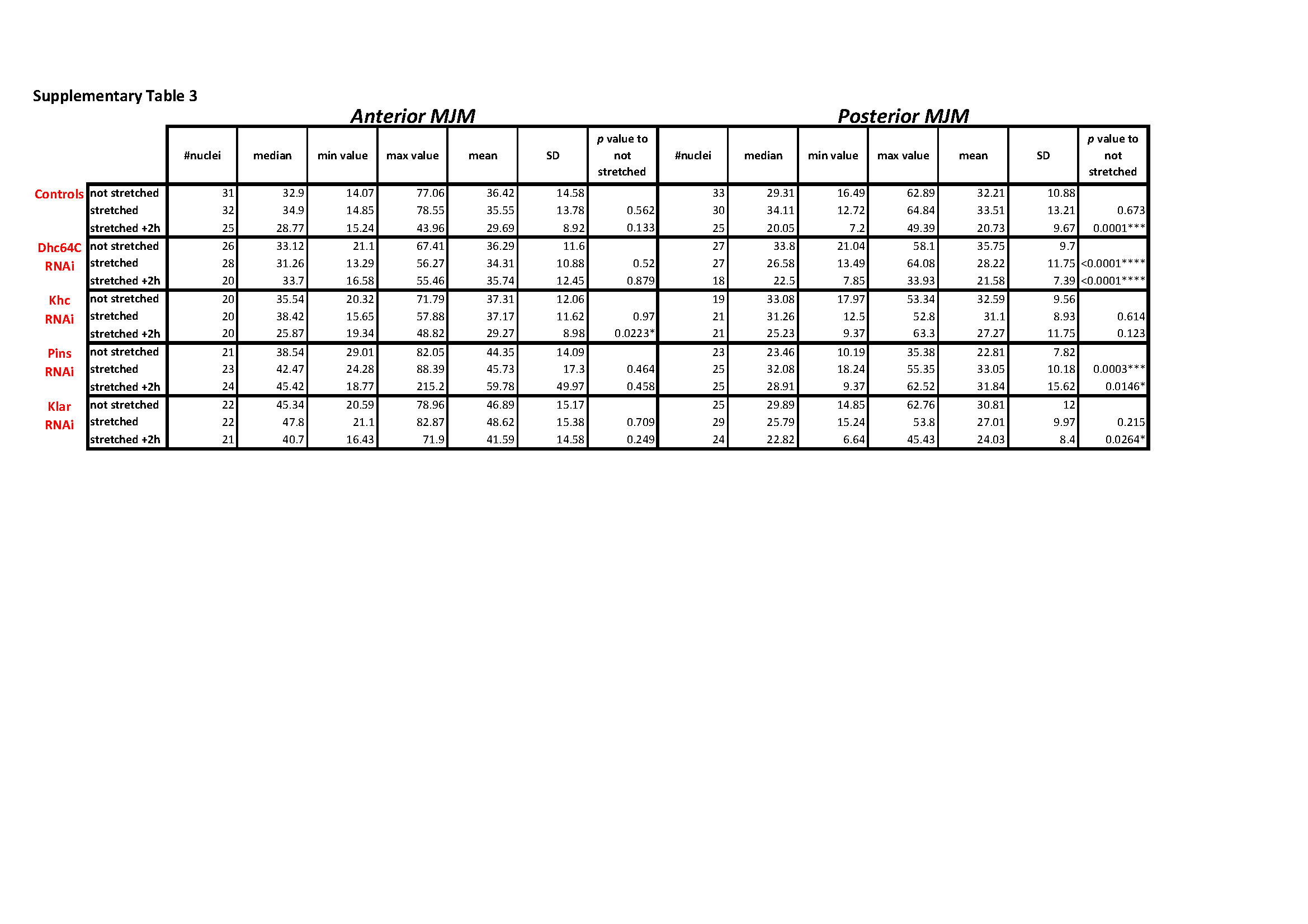

Supplement: Supplementary Table 3 — Statistical analysis for nuclear positioning in stretching experiments for segments A3 and A4. Student's t-test was used for comparison to unstretched muscles for each genotype. NP indicates that the non-parametric Mann–Whitney test. *p < 0.05, **p < 0.005, ***p < 0.0005, ****p < 0.0001. [file Image_4.TIFF]
